# Efavirenz-based simplification after successful early lopinavir-boosted-ritonavir-based therapy in HIV-infected children in Burkina Faso and Côte d’Ivoire: the MONOD ANRS 12206 non-inferiority randomised trial

**DOI:** 10.1186/s12916-017-0842-4

**Published:** 2017-04-24

**Authors:** Désiré Lucien Dahourou, Madeleine Amorissani-Folquet, Karen Malateste, Clarisse Amani-Bosse, Malik Coulibaly, Carole Seguin-Devaux, Thomas Toni, Rasmata Ouédraogo, Stéphane Blanche, Caroline Yonaba, François Eboua, Philippe Lepage, Divine Avit, Sylvie Ouédraogo, Philippe Van de Perre, Sylvie N’Gbeche, Angèle Kalmogho, Roger Salamon, Nicolas Meda, Marguerite Timité-Konan, Valériane Leroy, on behalf of the MONOD Study Group

**Affiliations:** 1 MONOD Project, ANRS 12206, Centre de Recherche Internationale pour la Santé, Ouagadougou, Burkina Faso; 2 Centre Muraz, Bobo-Dioulasso, Burkina Faso; 3 Inserm, Unité U1219, Université de Bordeaux, Bordeaux, France; 4 Paediatric Department, Centre Hospitalier Universitaire of Cocody, Abidjan, Côte d’Ivoire; 5 PACCI Programme, Site ANRS, Projet MONOD, Abidjan, Côte d’Ivoire; 6 Department of Infection and Immunity, Luxembourg Institute of Health, Luxembourg City, Luxembourg; 7 Laboratory CeDReS, Abidjan, Côte d’Ivoire; 8 Laboratory, Centre Hospitalier Universitaire de Ouagadougou, Ouagadougou, Burkina Faso; 9 EA 8, Université Paris Descartes, Paris, France; 10 Immunology, Hematology, Rhumatologie Unit, Hopital Necker-Enfants Malades–Assistance Publique–Hopitaux de Paris, Paris, France; 11 Paediatric Department, Centre Hospitalier Universitaire Yalgado Ouédraogo, Ouagadougou, Burkina Faso; 12 Paediatric Department, Centre Hospitalier Universitaire de Yopougon, Abidjan, Côte d’Ivoire; 13 Paediatric Department, Hôpital Universitaire des Enfants Reine Fabiola, Université Libre de Bruxelles, Brussels, Belgium; 14 Paediatric Department, Centre Hospitalier Universitaire Charles de Gaulle, Ouagadougou, Burkina Faso; 15 UMR 1058, Pathogenesis and control of chronic infections, Inserm/Université de Montpellier/EFS, Montpellier, France; 16 Department of Bacteriology-Virology, CHU Montpellier, Montpellier, France; 17 CePReF-enfants, Yopougon, Abidjan, Côte d’Ivoire; 18 University of Ouagadougou, Ouagadougou, Burkina Faso; 19 Inserm, Unité U1027, Université Toulouse 3, Toulouse, France

**Keywords:** Africa, HIV, Early antiretroviral treatment, Infants, Protease inhibitors, Lopinavir, Efavirenz, Randomised clinical trial, Virological outcomes, Treatment simplification

## Abstract

**Background:**

The 2016 World Health Organization guidelines recommend all children <3 years start antiretroviral therapy (ART) on protease inhibitor-based regimens. But lopinavir/ritonavir (LPV/r) syrup has many challenges in low-income countries, including limited availability, requires refrigeration, interactions with anti-tuberculous drugs, twice-daily dosing, poor palatability in young children, and higher cost than non-nucleoside reverse transcriptase inhibitor (NNRTI) drugs. Successfully initiating LPV/r-based ART in HIV-infected children aged <2 years raises operational challenges that could be simplified by switching to a protease inhibitor-sparing therapy based on efavirenz (EFV), although, to date, EFV is not recommended in children <3 years.

**Methods:**

The MONOD ANRS 12026 study is a phase 3 non-inferiority open-label randomised clinical trial conducted in Abidjan, Côte d’Ivoire, and Ouagadougou, Burkina Faso (ClinicalTrial.gov registry: NCT01127204). HIV-1-infected children who were tuberculosis-free and treated before the age of 2 years with 12–15 months of suppressive twice-daily LPV/r-based ART (HIV-1 RNA viral load (VL) <500 copies/mL, confirmed) were randomised to two arms: once-daily combination of abacavir (ABC) + lamivudine (3TC) + EFV (referred to as EFV) versus continuation of the twice-daily combination zidovudine (﻿ZDV) or﻿ ABC + 3TC + LPV/r (referred to as LPV). The primary endpoint was the difference in the proportion of children with virological suppression by 12 months post-randomisation between arms (14% non-inferiority bound, Chi-squared test).

**Results:**

Between May 2011 and January 2013, 156 children (median age 13.7 months) were initiated on ART. After 12–15 months on ART, 106 (68%) were randomised to one of the two treatment arms (54 LPV, 52 EFV); 97 (91%) were aged <3 years. At 12 months post-randomisation, 46 children (85.2%) from LPV versus 43 (82.7%) from EFV showed virological suppression (defined as a VL <500 copies/mL; difference, 2.5%; 95% confidence interval (CI), −11.5 to 16.5), whereas seven (13%) in LPV and seven (13.5%) in EFV were classed as having virological failure (secondary outcome, defined as a VL ≥1000 copies/mL; difference, 0.5%; 95% CI, −13.4 to 12.4). No significant differences in adverse events were observed, with two adverse events in LPV (3.7%) versus four (7.7%) in EFV (*p* = 0.43). On genotyping, 13 out of 14 children with virological failure (six out of seven EFV, seven out of seven LPV) had a drug-resistance mutation: nine (five out of six EFV, four out of seven LPV) had one or more major NNRTI-resistance mutations whereas none had an LPV/r-resistance mutation.

**Conclusions:**

At the VL threshold of 500 copies/mL, we could not conclusively demonstrate the non-inferiority of EFV on viral suppression compared to LPV because of low statistical power. However, non-inferiority was confirmed for a VL threshold of <1000 copies/mL. Resistance analyses highlighted a high frequency of NNRTI-resistance mutations. A switch to an EFV-based regimen as a simplification strategy around the age of 3 years needs to be closely monitored.

**Trial registration:**

ClinicalTrial.gov registry n°NCT01127204, 19 May 2010.

## Background

Despite effective interventions to prevent mother-to-child transmission (PMTCT) in sub-Saharan Africa [1], the seriousness of the paediatric epidemic remains real, mainly for operational reasons. According to UNAIDS, in 2013, 3.2 million children <15 years of age were living with HIV and 240,000 children were newly HIV-infected worldwide [2]. In the absence of antiretroviral therapy (ART), HIV-related infant mortality in Africa is dramatically high and occurs early, reaching 52% by the age of 2 years [3]. The 12-month efficacy of early ART initiated in all HIV-infected children reported in the CHER trial in South Africa showed a significant reduction of 76% in infant mortality among children treated immediately from 12 weeks of age, compared to those deferred according to the 2006 World Health Organization (WHO) recommendations [4]. Consequently, ART initiation was recommended for all HIV-infected children <12 months of age in 2008 [5], extended to all children <24 months in 2010 [6], and at the earliest convenience in all those <5 years in 2013 [7]. In 2015, WHO recommended that ART be initiated in everyone living with HIV at any CD4 cell count [8].

In low-income countries, the first-line therapy recommended for all children <36 months is based on a boosted protease inhibitor, lopinavir-boosted ritonavir (LPV/r), regardless of perinatal non-nucleoside reverse transcriptase inhibitor (NNRTI) exposure [7, 8]. Two trials have demonstrated the superiority of first-line LPV/r-based ART compared to first-line nevirapine (NVP) in populations of young children with or without pre-exposure to NVP for PMTCT [9, 10]. LPV/r is a potent drug, with a high genetic barrier against resistance, and is especially effective when combined with two nucleoside reverse transcriptase inhibitors (NRTIs) [11]. First-line LPV/r also leads to longer life expectancy and is cost saving compared to first-line NVP [12]. The backbone recommended is based on two NRTIs: abacavir (ABC), preferentially, or zidovudine (AZT), and lamivudine (3TC).

The choice of LPV/r-based therapy may nevertheless be operationally challenging, with many drawbacks restraining its use as a first-choice therapy in young children in Africa. The currently available oral syrup forms of LPV/r for infants have a thermostability issue that requires the use of refrigeration for storage and distribution [13], as well as having poor palatability. LPV/r is also used as a second-line drug owing to the current scarcity of antiretroviral drugs adapted to paediatric use. In addition, there are potential metabolic complications and interactions with anti-tuberculous drugs [14]. In May 2015, the United State Food and Drug Administration (FDA) approved the use of LPV/r oral pellets in children (http://www.accessdata.fda.gov/drugsatfda_docs/appletter/2015/205425Orig1s000TAltr.pdf), which has overcome the challenges of the cold chain. However, these pellets still need to be assessed in Africa. Consequently, we explored whether it would be possible to substitute this initial LPV-based regimen in children with confirmed virological suppression with a once-daily ART that is easier to handle and more acceptable, while saving protease inhibitors for later use if virological failure occurs, in the context of poor access to second-line treatments. We selected efavirenz (EFV) dosed once daily with paediatric-friendly formulations, which is well tolerated and can be used in combination with anti-tuberculous drugs [15].

We hypothesised that, for children who were started on a twice-daily LPV-based ART and who were virologically suppressed after an initial 12–15-month period, ART could be simplified in the long term with a once-daily EFV-based therapy.

## Methods

### Study design

The MONOD ANRS 12206 study is a non-inferiority, open-label phase 3 randomised clinical trial conducted in Ouagadougou, Burkina Faso, and Abidjan, Côte d’Ivoire (ClinicalTrial.gov registry number: NCT01127204, first registered on 19 May 2010). Study sites were the Abobo-Avocatier urban health clinic, the CePReF-enfant and the Yopougon and Cocody University Hospitals in Abidjan, and the Yalgado Ouédraogo and the Charles de Gaulle University Hospitals in Ouagadougou. The protocol was approved by the Comité d’Ethique pour la Recherche en Santé du Burkina Faso and the Comité National d’Ethique et de la Recherche en Côte d’Ivoire.

### Participants

An initial therapeutic cohort included all children with an HIV-1 infection confirmed by HIV-1 DNA polymerase chain reaction (PCR) who were aged <24 months, free of tuberculosis, and antiretroviral-naïve except for exposure to PMTCT interventions. Both parents had to provide written consent. This initial cohort received 12–15 months of treatment with two NRTIs (ABC or AZT and 3TC) and LPV/r given twice daily, together with prophylaxis against opportunistic infections with cotrimoxazole and therapeutic education. Exclusion criteria were age ≥24 months; on current ART; a known intolerance to at least one of the drugs; HIV-2-infected or HIV-1 and -2 co-infected; tuberculosis; or a haemoglobin level <7 g/dL, neutrophils ≤750/mm^3^, creatinine ≥5× normal range, or aspartate transaminase (AST) or alanine transaminase (ALT) ≥5× normal range. After the initial cohort period, children with an undetectable HIV-1 RNA viral load (VL) <500 copies/mL at 12 months (confirmed at a 3-month interval) were randomised to either switch to once-daily ABC + 3TC + EFV (hereafter referred to as EFV) therapy or stay on the twice-daily LPV regimen (AZT + 3TC + LPV/r or ABC + 3TC + LPV/r). Children with a detectable VL were not randomised and were maintained on a LPV regimen with therapeutic education reinforcement.

### Randomisation

A centralised computer-generated sequentially numbered block randomisation list, stratified according to country, was drawn up and included in an online software randomisation system to allocate the treatment arm through a secure website set up by the data management centre in Bordeaux, France. After the programme verified all pre-specified inclusion criteria, children were automatically randomly assigned to one arm (1:1), either the control strategy (LPV-based therapy) the simplified strategy (EFV-based therapy). After randomisation, an automatic printout showing the treatment decision and the ID number of the randomised arm was forwarded to the trial coordinator.

### Trial treatments

In the control strategy, children received twice-daily triple therapy: (ZDV) (syrup 10 mg/mL, 4 mg/kg every 12 hours) or ABC (syrup 20 mg/mL, 8 mg/kg every 12 hours) + 3TC (syrup 10 mg/mL, 4 mg/kg every 12 hours) + LPV/r (syrup 80/20 mg/mL, 12 mg/kg every 12 hours). In the simplified strategy, children received once-daily triple therapy: ABC (syrup 20 mg/mL, 16 mg/kg every morning) + 3TC (syrup 10 mg/mL, 8 mg/kg every morning) + EFV (syrup 30 mg/mL, 25 mg/kg every morning on an empty stomach). Although EFV is not recommended for children <3 years or <10 kg, according to the WHO guidelines [7], we used EFV in children younger than the recommended age and at a dosage of 25 mg/kg, according to a paediatric pharmacokinetic (PK) study conducted in Burkina Faso [16]. As recommended by the WHO, all children systematically received prophylaxis for opportunistic infections with cotrimoxazole syrup: sulfamethoxazole (20 mg/kg) + trimethoprim (4 mg/kg) once daily during the entire study. The assistant pharmacist and social worker systematically delivered therapeutic education when the drugs were given to families.

The drugs were provided by the national AIDS programmes under the responsibility of the country coordinating centres in charge of supplies and qualification of the batches. The inclusion process in the initial cohort started in May 2011. In 2012, in Abidjan, the national AIDS control programme introduced oro-dispersible fixed-dose formulation tablets for ABC and 3TC using WHO weight band dosing to substitute for the syrup formulations.

### Procedures

A pre-inclusion visit, 4 weeks before ART initiation, included informed consent, an interview to assess medical history (perinatal or neonatal PMTCT exposure), a complete clinical examination (weight, height and WHO clinical staging), tuberculosis screening (chest X-ray), a standard blood test (haematology, creatinine, urea, AST, ALT, total bilirubin, glucose, alkaline phosphatases, amylase, lipase, lipid assessment [total cholesterol, triglycerides, low-density lipoprotein, high-density lipoprotein, very low-density lipoprotein], fasting blood glucose), a CD4 lymphocyte sub-population count (percentage and absolute count), and a confirmation of HIV status (quantitative HIV-1 RNA in plasma and quantitative proviral DNA). Where symptoms suggested tuberculosis (prolonged fever, chronic cough, recent malnutrition or failure of classic antibiotics for an infectious syndrome) the diagnosis was completed with a tuberculin skin test, gastric lavage on three consecutive days and stool examination for *Mycobacterium tuberculosis*.

All children were followed for 12–15 months after inclusion (defined as ART initiation), then for 12 months after randomisation. After inclusion, children had monthly clinical follow-up visits recording all clinical events, including adverse effects, weight- and height-for-age z-scores calculated using the WHO software, drug uptake, measurement of adherence (doses taken in the past 4 days), delivery of drugs and therapeutic education. Screening for clinical neurological/sleep adverse events was performed at each monthly visit by trained paediatricians who systematically looked for sleeping adverse events and performed a neurological examination.

Standard blood tests were repeated every 6 months. CD4 cell counts and VL were measured quarterly at the Laboratoire du CeDreS in Abidjan and the Reference Laboratory of CHU Charles de Gaulle in Ouagadougou using a FACScan flow cytometer (Becton Dickinson, Mountain View, CA). VLs were measured with real-time PCR using a commercial assay (Generic HIV Charge Virale, Biocentric, Bandol, France) [17]. These methods were being used at that time according to the manufacturer’s protocol in both Abidjan and Ouagadougou, where this method was validated. At the time of study implementation, in 2011, the manufacturer’s threshold for VL was 400 copies/mL as written in the protocol, but the threshold validated at the country level differed: it was 400 copies/mL in the Abidjan laboratory and 500 copies/mL in the Ouagadougou laboratory. The laboratory in Ouagadougou was not able to guarantee results below 500 copies/mL. Therefore, we decided to homogenise the threshold to <500 copies/mL at both sites to define viral suppression. HIV-1 genotypic resistance testing was performed upon virological failure (HIV-1 RNA ≥1000 copies/mL, the commonly used threshold to guide treatment strategies [8]) and at enrolment before ART initiation in Abidjan for samples collected in Côte d’Ivoire and in Luxembourg for samples collected in Burkina Faso. The ANRS consensus technique (www.hivfrenchresistance.org) was used to genotype protease and reverse transcriptase genes. Sequences were edited with Bio-Edit sequence Alignment Editor (version 7.0) and trees constructed with Mega 4. Relevant drug-resistance mutations were interpreted according to the Stanford University HIV Drug Resistance Database (HIVdb Program, http://hivdb.stanford.edu) and the ANRS-v24 interpretation rule (http://www.hivfrenchresistance.org/2011/Algo-2011.pdf). HIV-1 subtypes were assigned using REGA (http://www.bioafrica.net/rega-genotype/html/index.html) and COMET (http://comet.retrovirology.lu) HIV-1 subtyping tools against reference HIV-1 group M sequences from GenBank (http://www.ncbi.nlm.nih.gov/Genbank/index.html). Blood samples were collected from children and transferred for processing within 4 hours. Two plasma samples were prepared and stored at −80 °C: one to perform VL measurement and one for resistance testing if the VL was >1000 copies/mL. Quality controls were performed for diagnostic PCR and VL (CDC, Atlanta, GA, USA) every 6 months and for genotyping (ANRS, Paris, France or Quality Controls in Molecular Diagnostics, Utrecht, the Netherlands) on a yearly basis.

### Outcomes

The primary outcome was the proportion of children alive and with virological suppression (defined as HIV-1 RNA <500 copies/mL) at 12 months post-randomisation; this specific time point was considered regardless of any detectable VL between randomisation and 12 months. Secondary outcomes were virological failure (defined as HIV-1 RNA ≥1000 copies/mL, the commonly used threshold to guide treatment strategies [8]) at 12 months post-randomisation, adverse events, resistance mutation profiles, the clinical-immunological response, the pharmacokinetic parameters, adherence, and cost.

### Statistical analysis

For a non-inferiority trial, the statistical parameter of interest is the difference in successful viral suppression rate 12 months after the switch, defined in this instance as the rate of viral suppression in the LPV arm (control) minus the rate in the EFV arm, using a Chi-squared test. If this difference is >0, outcomes favour the control group [18]. We aimed to obtain virological success of at least 76% at 12 months post-switch. We pre-specified that a margin of <14% for the 95% confidence interval (CI) of the difference in the primary outcome between the two arms would meet our criteria for non-inferiority. Both an intention-to-treat analysis conducted using all available data, and a per-protocol analysis were conducted as recommended for non-inferiority trials [19]. Based on our anticipated enrolment of 146 children with 73 children per arm, we expected an 80% power to detect this difference. In the CHER trial, the 12-month survival probability in infants on a LPV/r-based triple therapy was 96% [4]. Because virological data were not yet available at the time of our protocol, we expected a 95% response on LPV according to the Yeni 2008 report [11]. Thus, assuming a 12-month virological suppression among survivors on LPV of 90%, we anticipated recruited 162 children in the initial LPV-based cohort. To compare the characteristics of the study population, we used Chi-squared or Fisher’s exact tests for categorical variable and *t* tests or Mann–Whitney tests for continuous variables. We analysed the correlates of viral suppression at 12 months post-randomisation, using a multivariate logistic regression. All *p* values were two sided and *p* < 0.05 was considered statistically significant. Analyses were performed using SAS version 9.1.3.

## Results

### Trial profile and baseline characteristics

**Fig. 1:**
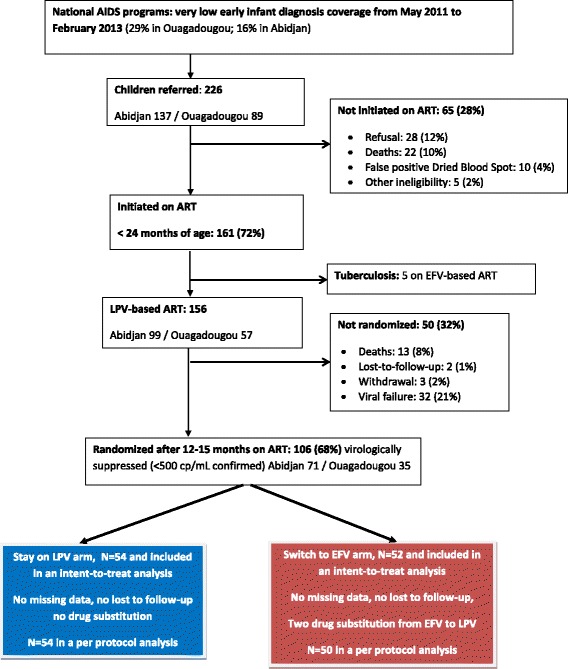
MONOD ANRS 12206 Trial profile in Abidjan, Côte d’Ivoire, and Ouagadougou, Burkina Faso, 2011–2015. *ART* antiretroviral therapy, *EFV* efavirenz-based ART, *LPV* lopinavir-boosted-based ART

Between May 2011 and January 2013, 226 children were referred to the study clinics (Fig. 1). Of these, 65 children (28%) were not initiated on ART, mainly due to parent’s refusal (12%), early deaths (10%), a false-positive dried blood spot HIV DNA PCR result (4%) or other reasons (2%). That left 161 children (72%) who were initiated on ART before 24 months of age [20]. Among them, five were initiated on EFV-based ART because of tuberculosis co-infection at inclusion.

The remaining 156 children were initiated on LPV-based ART (Fig. 1). Their median age at HIV-1 diagnosis was 8.5 months, and at ART initiation was 13.7 months. After 12–15 months on ART, only 68% were alive and showed virological suppression: 13 had died (8%), two were lost to follow-up (1%), three withdrew (2%) and 32 had virological failure (21%). Details on this cohort are presented elsewhere [21].

Of the 106 children who were eligible for randomisation, that is, alive and showing virological suppression, 54 were randomised to maintain LPV therapy, and 52 to switch to EFV (Fig. 1); all were included in the intention-to-treat analysis. Among the children randomised, 91% (97 out of 106) were aged <3 years (49 in the LPV arm and 48 in the EFV arm).

There were no significant differences between the two groups’ baseline characteristics at the time of randomisation (Table 1). Overall, 67.0% lived in Abidjan, 55.7% were girls, the father was the main caregiver for 17.0%, 39.6% had not been exposed to any PMTCT intervention or maternal ART, 30.2% were exposed to perinatal PMTCT prophylaxis alone, 8.5% were born to mothers on ART, and 21.7% were exposed to postnatal maternal ART initiated during breastfeeding (Table 1). At the time of ART initiation, the children already had advanced HIV-disease progression: 54.7% were WHO stage 3 or 4 [6], the median CD4 percentage was 20.8% and their mean VL was 6.1 log10 copies/mL (SD: 1). After 12–15 months on ART, at the time of randomisation, the median age was 26.8 months and median CD4% had increased to 35.9%; the CD4% for both groups was within the normal range. Overall, children were virologically suppressed for a median of 6 months before randomisation.

**Table 1:** Baseline characteristics according to randomisation arm of the 106 HIV-1-infected children randomised in the ANRS 12206 MONOD trial (Abidjan and Ouagadougou, May 2011–April 2014)

Characteristics	Total N = 106	AZT or ABC + 3TC + LPV/r (twice daily) N = 54	ABC + 3TC + EFV (once daily) N = 52	*p* value
Pre-trial characteristics				
Abidjan site, n (%)	71 (67.0)	36 (66.7)	35 (67.3)	0.94
Age (months) at HIV-1 diagnosis, median (IQR)	8. 5 (3.3–15.6)	8.4 (3.8–16.5)	9.8 (2.8–15.4)	0.84
Age (months) at ART initiation, median (IQR)	13.7 (7.9–18.4)	12.8 (8.1–18.4)	14.2 (7.6–18.4)	0.96
Female, n (%)	59 (55.7)	35 (64.8)	24 (46.2)	0.05
Father or other as main caregiver, n (%)	18 (17.0)	10 (18.5)	8 (15.4)	0.67
Tap water at home, n (%)	78 (73.6)	39 (72.2)	39 (75.0)	0.74
Electricity at home, n (%)	84 (79.2)	43 (79.6)	41 (78.9)	0.92
Ever breastfed from birth, n (%)	92 (86.8)	45 (83.3)	47 (90.4)	0.28
Breastfeeding duration (months) for those breastfed, median (IQR)	13.8 (7.6–21.4)	16.0 (7.5–21.5)	12.0 (7.7–19.6)	0.49
History of antiretroviral drug exposure				
Prenatal maternal ART, n (%)	9 (8.5)	5 (9.3)	4 (7.7)	1.00
AZT/TDF + 3TC/FTC + NVP	8 (88.9)	4 (80.0)	4 (100.0)	
AZT + 3TC + EFV	1 (11.1)	1 (20.0)	0 (0.0)	
PMTCT and postnatal maternal ART	10 (9.4)	7 (13.0)	3 (5.8)	0.32
PMTCT				1.00
sdNVP-based PMTCT	2 (20.0)	2 (28.6)	0 (0.0)	
Other than sdNVP-based PMTCT	8 (80.0)	5 (71.4)	3 (100.0)	
Postnatal maternal HAART				1.00
AZT/TDF + 3TC/FTC + NVP	8 (80.0)	5 (71.4)	3 (100.0)	
AZT + 3TC + LPV/r	2 (20.0)	2 (28.6)	0 (0.0)	
PMTCT only	32 (30.2)	15 (27.8)	17 (32.7)	0.58
Only sdNVP-based PMTCT	5 (15.6)	3 (20.0)	2 (11.8)	
Other than sdNVP-based PMTCT	27 (84.4)	12 (80.0)	15 (88.2)	
Postnatal maternal ART only	13 (12.3)	7 (13.0)	6 (11.5)	0.82
D4T/AZT + 3TC + NVP	8 (61.5)	4 (57.1)	4 (66.7)	
AZT/D4T/TDF + 3TC/FTC + EFV	3 (23.1)	2 (28.6)	1 (16.7)	
D4T + 3TC + LPV/r	1 (7.7)	0 (0.0)	1 (16.7)	
Missing	1 (7.7)	1 (14.3)	0 (0.0)	
No previous exposure to any PMTCT or maternal ART	42 (39.6)	20 (37.0)	22 (42.3)	0.58
Z-scores at child’s ART initiation, mean (SD)				
Weight-for-age	−2.3 (1.5)	−2.3 (1.4)	−2.3 (1.6)	0.81
Height-for-age	−2.2 (1.7)	−2.1 (1.7)	−2.3 (1.7)	0.53
Weight-for-height	−1.5 (1.4)	−1.5 (1.3)	−1.4 (1.5)	0.84
WHO stage				0.86
Stage 1 or 2, n (%)	48 (45.3)	24 (44.4)	24 (46.2)	
Stage 3 or 4, n (%)	58 (54.7)	30 (55.6)	28 (53.9)	
Haemoglobin (g/dL), median (IQR)	9.2 (8.4–9.9)	9.1 (8.5–10.0)	9.4 (8.4–9.9)	0.75
CD4 %, median (IQR)	20.8 (14.2–28.1)	18.9 (13.9–27.4)	21.2 (15.0–28.8)	0.65
Viral load (log_10_ copies/mL), median (SD)	6.1 (1.0)	6.2 (1.0)	6.0 (1.0)	0.51
Viral load ≥6 log_10_ copies/mL, n (%)	58 (54.7)	30 (55.6)	28 (53.8)	0.86
First-line NRTI backbone				0.31
ZDV-3TC, n (%)	95 (89.6)	50 (92.6)	45 (86.5)	
ABC-3TC, n (%)	11 (10.4)	4 (7.4)	7 (13.5)	
Ever start cotrimoxazole, n (%)	104 (98.1)	53 (98.1)	51 (98.1)	1.00
At randomisation				
Age (months), median (IQR)	26.8 (21.5–31.5)	26.0 (21.8–31.3)	27.2 (20.8–31.5)	0.84
Duration on HAART (months), median (IQR)	12.7 (12.1–13.0)	12.7 (12.1–13.0)	12.6 (12.1–13.0)	0.86
Weight (kg), median (IQR)	10.2 (9.2–11.4)	10.2 (9.3–11.2)	10.2 (9.1–11.6)	0.92
WHO stage 3 or 4, n (%)	49 (46.2)	25 (46.3)	24 (46.1)	0.99
CD4 %, median (IQR)	35.9 (28.5–40.9)	36.4 (28.5–40.7)	34.9 (28.5–41.1)	0.63
On cotrimoxazole, n (%)	106 (100.0)	54 (100.0)	52 (100.0)	–

*AZT* Zidovudine, *ABC* Abacavir, *3TC* Lamivudine, *LPV/r* Lopinavir-boosted ritonavir, *EFV* Efavirenz, *IQR* Interquartile range, *ART* Antiretroviral therapy, *TDF* Te﻿nofovir, *FTC* Emtr﻿icitabine, *NVP* Nevirapine, *PMTCT* Prevention of mother-to-child-transmission, *sdNVP* Single-dose nevirapine, *HAART* Highly active antiretroviral therapy, *D4T* Stavudine, *SD* Standard deviation, *WHO* World Health Organization, *NRTI* Nucleoside reverse transcriptase inhibitor

### Virological suppression

At 12 months post-randomisation, all children were alive and followed up, without any missing data on VL outcomes (Table 2). In an intention-to-treat analysis, 46 out of 54 children (85.2%) in the LPV arm vs. 43 out of 52 (82.7%) in the EFV arm had a VL <500 copies/mL (*p* = 0.72). The difference was 2.5% (95% CI, −11.5 to 16.5), tending to favour the LPV arm. The 95% CI included the pre-specified non-inferiority margin of 14%; therefore, this analysis was deemed inconclusive. The actual statistical power to detect a difference was 67%. Among the children aged <3 years, 42 out of 49 (85.7%) in the LPV arm vs. 39 out of 48 (81.3%) in the EFV arm had a VL <500 copies/mL (*p* = 0.55).

**Table 2:** Twelve-month post-randomisation primary and secondary outcomes in the 106 HIV-1-infected children randomised in the ANRS 12206 MONOD study according to arm (Abidjan and Ouagadougou, February 2013–April 2015)

12-month outcomes	Total *N* = 106	Arm 1: AZT + 3TC + LPV/r (twice daily) *N* = 54	Arm 2: ABC + 3TC + EFV (once daily) N = 52	*p* value
Follow-up (months), median (IQR)	12.7 (12.1–13.0)	12.7 (12.1–13.0)	12.6 (12.1–13.0)	0.44
Death	0 (0.0)	0 (0.0)	0 (0.0)	-
Loss to follow-up	0 (0.0)	0 (0.0)	0 (0.0)	-
Withdrawal	0 (0.0)	0 (0.0)	0 (0.0)	-
Virological success (VL < 500 copies/mL)	89 (84.0)	46 (85.2)	43 (82.7)	0.72
Virological failure (500 ≥ VL < 1000 copies/mL)	3 (2.8)	1 (1.8)	2 (3.8)	-
Virological failure (VL ≥ 1000 copies/mL)	14 (13.2)	7 (13.0)	7 (13.5)	0.5﻿9
CD4 %, median (IQR)	37.3 (31.6–41.9)	37.3 (31.3–41.6)	37.1 (31.6–42.0)	0.85
Immunodeficiency for age^a^				0.59
None	57 (53.8)	32 (59.3)	25 (48.1)	
Mild	38 (35.9)	17 (31.5)	21 (40.4)	
Severe	3 (2.8)	2 (3.7)	1 (1.9)	
Missing	8 (7.6)	3 (5.6)	5 (9.6)	
Z-score, mean (SD)				
Weight-for-age	−1.2 (0.9)	−1.3 (0.8)	−1.2 (1.0)	0.63
Height-for-age	−1.4 (1.1)	−1.5 (1.1)	−1.4 (1.2)	0.84
Weight-for-height	−0.6 (0.8)	−0.6 (0.9)	−0.5 (0.8)	0.62

*AZT* Zidovudine, *ABC* Abacavir, *3TC* Lamivudine, *LPV/r* Lopinavir-boosted ritonavir, *EFV* Efavirenz, *IQR* Interquartile range, *VL* Viral load, *SD* Standard deviation

^a^Severe immunodeficiency for age: CD4 < 25% if aged <2 years, CD4 < 20% if aged ≥2 years; mild immunodeficiency for age: CD4 between 25 and 35% if aged <2 years, CD4 between 20 and 35% if aged ≥2 years; No immunodeficiency for age if CD4 > 35%

With regards our secondary outcome of virological failure using the threshold of >1000 copies/mL, in the intention-to-treat analysis, 7 out of 54 children (13.0%) in the LPV arm failed vs. 7 out of 52 (13.5%) in the EFV arm (*p* = 0.59). The difference between these rates was −0.5% (95% CI, −13.4 to 12.4). The 95% CI does not include the pre-specified non-inferiority margin of 14%; therefore, at the 1000 copies/mL threshold, EFV was considered non-inferior to LPV in our trial.

There were no significant differences in children’s characteristics according to virological success (Tables 3 and 4). For the 17 children who failed to show virological suppression (≥500 copies/mL) at 12 months, 10 failures occurred within the first 6 months. Drug modifications occurred in two children, who were switched from EFV to LPV: one for sleeping disorders persisting 10 months after randomisation, and one for hyper-transaminasaemia due to a cytotoxic treatment administrated by a healer (Table 5). The sensitivity per-protocol analysis gave similar results compared to the intention-to-treat analysis: 46 children (85.2%) in the LPV arm vs. 42 (84.0%) in the EFV arm had a VL <500 copies/mL, a difference of 1.2% (95% CI, −12.7 to 15.1). Using the 1000 copies/mL threshold, seven children (13.0%) in the LPV arm showed virological suppression vs. six (12.0%) in the EFV arm, with a difference of one (95% CI, −11.7 to 13.7).

**Table 3:** Factors associated with 12-month virological success (<500 copies/mL) in the 106 HIV-1-infected children randomised in the ANRS 12206 MONOD study (Abidjan and Ouagadougou, February 2013–February 2015)

	Total *N* = 106	Virological success (<500 copies/mL) *N* = 89	Virological failure (≥500 copies/mL) *N* = 17	*p* value
Country				0.83
Abidjan	71 (67.0)	60 (67.4)	11 (64.7)	
Ouagadougou	35 (33.0)	29 (32.6)	6 (35.3)	
Sex				0.81
Female	59 (55.7)	50 (56.2)	9 (52.9)	
Male	47 (44.3)	39 (43.8)	8 (47.1)	
Treatment arm				0.73
AZT/ABC + 3TC + LPV/r	54 (50.9)	46 (51.7)	8 (47.1)	
ABC + 3TC + EFV	52 (49.1)	43 (48.3)	9 (52.9)	
Main caregiver for children				1.00
Mother main caregiver	88 (83.0)	74 (83.2)	14 (82.4)	
Father/other in charge of care	18 (17.0)	15 (16.8)	3 (17.6)	
Father informed of the child’s HIV status				0.30
No	44 (41.5)	35 (39.3)	9 (52.9)	
Yes	62 (58.5)	54 (60.7)	8 (47.1)	
History of antiretroviral drug exposure				0.63
Prenatal maternal ART	9 (8.5)	7 (7.9)	2 (11.8)	
PMTCT only	32 (30.2)	27 (30.3)	5 (29.4)	
Postnatal maternal ART only	13 (12.3)	12 (13.5)	1 (5.9)	
PMTCT and postnatal maternal ART	10 (9.4)	7 (7.9)	3 (17.6)	
No previous exposure to any PMTCT or ART	42 (39.6)	36 (40.4)	6 (35.3)	
Age at randomisation				0.39
<24 months	41 (38.7)	36 (40.4)	5 (29.4)	
≥24 months	65 (61.3)	53 (59.6)	12 (70.6)	
WHO clinical stage at randomisation				
Stage 1, 2, 3	83 (78.3)	73 (82.0)	10 (58.8)	0.05
Stage 4	23 (21.7)	16 (18.0)	7 (41.2)	
Z-score Weight-for-age at ART initiation				0.35
Normal	50 (47.2)	44 (49.4)	6 (35.3)	
Moderate	22 (20.7)	19 (21.4)	3 (17.6)	
Severe	34 (32.1)	26 (29.2)	8 (47.1)	
Z-score Height-for-age at ART initiation				0.09
Normal	55 (51.9)	50 (56.2)	5 (29.4)	
Moderate	21 (19.8)	15 (16.8)	6 (35.3)	
Severe	30 (28.3)	24 (27.0)	6 (35.3)	
CD4 % at ART initiation				0.60
>35%	13 (12.3)	10 (11.2)	3 (17.6)	
25–35%	19 (17.9)	17 (19.1)	2 (11.8)	
<25% or missing	74 (69.8)	62 (69.7)	12 (70.6)	

Data are presented as n (%)

*AZT* Zidovudine, *3TC* Lamivudine, *ABC* Abacavir, *EFV* Efavirenz, *LPV/r* Lopinavir-boosted ritonavir, *ART* Antiretroviral therapy, *PMTCT* Prevention of mother-to-child-transmission, *WHO* World Health Organization. ﻿Normal: Z-score ≥ 2 Standard Deviations (SD); Z-score <-2 SD corresponds to﻿ moderate malnutrition, being severe form at a Z-score<-3 SD

**Table 4:** Factors associated with 12-month virological success (<500 copies/mL) in the 106 HIV-1-infected children randomised in the ANRS 12206 MONOD study (Abidjan and Ouagadougou, February 2013–February 2015): logistic regression

	Univariate			Adjusted model^a^		
	OR	CI (95%)	*p* value	aOR	CI (95%)	*p* value
Abidjan vs. Ouagadougou	1.13	(0.38–3.35)	0.83	0.64	(0.17–2.41)	0.51
Female vs. Male	1.14	(0.40–3.23)	0.81	1.26	(0.41–3.93)	0.68
Treatment arm			0.73			0.66
AZT/ABC + 3TC + LPV/r	Ref.	–		Ref.	–	
ABC + 3TC + EFV	0.83	(0.29–2.35)		0.78	(0.26–2.38)	
Mother main caregiver vs. father or other	1.06	(0.27–4.14)	0.94	–	–	
Father informed of HIV status of the child	1.74	(0.61–4.93)	0.30	–	–	
History of antiretroviral drug exposure			0.68			0.34
No previous exposure to any PMTCT or ART	Ref.	–		Ref.	–	
Prenatal maternal ART	0.58	(0.10–3.51)		0.32	(0.04–2.32)	
Exposure to PMTCT only	0.90	(0.25–3.26)		0.64	(0.15–2.68)	
Exposure to postnatal maternal ART only	2.00	(0.22–18.33)		1.95	(0.20–19.24)	
PMTCT and postnatal maternal ART	0.39	(0.08–1.94)		0.19	(0.03–1.21)	
Age at randomisation <24 months	1.63	(0.53–5.03)	0.39	–	–	
WHO clinical stage at randomisation			0.04			0.01
Stage 1, 2, 3	Ref.	–		Ref.	–	
Stage 4	0.31	(0.10–0.95)		0.18	(0.05–0.72)	

*OR* Odds ratio, *CI* Confidence interval, *aOR* Adjusted odds ratio, *AZT* Zidovudine, *3TC* Lamivudine, *LPV/r* Lopinavir-boosted ritonavir, *ABC* Abacavir, *EFV* Efavirenz, *PMTCT* Prevention of mother-to-child-transmission, *ART* Antiretroviral therapy, *WHO* World Health Organization

^a^Forced variables: country, sex, treatment arm and history of antiretroviral drug exposure

**Table 5:** Incidence of post-randomisation grade 3 and 4 adverse events in the 106 HIV-1-infected children randomised in the ANRS 12206 MONOD study according to arm (Abidjan and Ouagadougou, February 2013–April 2015)

Outcomes	Total *N* = 106	Arm 1: AZT/ABC + 3TC + LPV/r (twice daily) N = 54	Arm 2: ABC + 3TC + EFV (once daily) N = 52	*p* value
SAE				
Hospitalisations and clinical SAE	6 (5.7)	2^d^ (3.7)	4^e^ (7.7)	0.43
Grade 3 or 4 adverse events^a^	1 (0.9)	0 (0.0)	1 (1.9)	0.90
Toxicity causing ART modification^b^	3 (2.8)	1 (1.9)	2 (3.8)	0.61
Sleeping disorders declared by caregivers	9 (8.5)	4 (7.4)	5 (9.6)	0.74
Specific biological adverse events^c^				
Anaemia, grade 3 and 4	3 (2.8)	1 (1.9)	2 (3.8)	0.61
Neutropenia, grade 3 and 4	10 (9.4)	9 (16.7)	1 (1.9)	0.02
Thrombopenia, grade 3 and 4	1 (0.9)	1 (1.9)	0 (0.0)	1.00
Hyperglycaemia, grade 3 and 4	0 (0.0)	0 (0.0)	0 (0.0)	-
Hypercholesterolemia, grade 3	0 (0.0)	0 (0.0)	0 (0.0)	-
Hypertriglyceridemia, grade 3 and 4	0 (0.0)	0 (0.0)	0 (0.0)	-
Hypercreatininaemia, grade 3 and 4	0 (0.0)	0 (0.0)	0 (0.0)	-
Hypertransaminasaemia AST or ALT, grade 3 and 4	2 (1.9)	1 (1.9)	1 (1.9)	1.00
Hyperbilirubinaemia, grade 3 and 4	5 (4.7)	3 (5.6)	2 (3.9)	1.00
Hyperamylasaemia, grade 3 and 4	2 (1.9)	0 (0.0)	2 (3.9)	0.24
Hyperlipasaemia, grade 3 and 4	0 (0.0)	0 (0.0)	0 (0.0)	-

Data are presented as n (%)

*AZT* Zidovudine, *3TC* Lamivudine, *LPV/r* Lopinavir-boosted ritonavir, *ABC* Abacavir, *EFV* Efavirenz, *SAE* Serious adverse events, *ART* Antiretroviral therapy, *AST* Aspartate transaminase*, ALT* Alanine transaminase

^a^Hepatitis due to a cytotoxic treatment administrated by a healer

^b^One toxicity substitution in a child randomised to LPV/r was from AZT to ABC for neutropenia. Two toxicity substitutions in children randomised to EFV to LPV arm: one for sleeping disorders persisting 10 months after randomisation and one for hypertransaminasaemia due to a cytotoxic treatment administrated by a healer

^c^No other biological SAE including glycaemia, cholesterolaemia, triglyceridaemia, creatininaemia, lipaseamia

^d^2 gastroenteritis

^e^1 gastroenteritis, 1 pneumonia, 1 upper respiratory infection with malaria, 1 malaria

### Other secondary outcomes

After 12 months, 53.8% of children overall were non-immunodeficient, and there were no significant differences in CD4% between arms: the median CD4% was 37.3% in the LPV arm vs. 37.1% in the EFV arm (*p* = 0.85; Table 2). Children in the LPV arm had a 0.1 lower mean z-score for weight-for-age, height-for-age and weight-for-height, although this difference was not significant between arms. There were no significant differences in the occurrence of severe adverse events after randomisation (Table 5): two hospitalisations occurred in the LPV arm vs. four in the EFV arm, all due to infectious diseases (*p* = 0.43). One grade 4 adverse event occurred in the EFV arm, with hepatitis due to cytotoxic treatment administered by a healer, but this was not judged as antiretroviral-related. There were no significant differences in day-time or night-time sleeping disorders declared by caregivers between the arms, with four in the LPV arm vs. five in the EFV arm, though one child in the EFV arm did have persistent day-time and night-time sleeping disorders leading to a treatment substitution to LPV after 10 months. No clinical seizures were reported. There was no difference in the number of higher grade (grade 3 or 4) biological adverse events between the EFV and LPV arms, although we noted a significantly higher rate of neutropenia in the LPV arm, often associated with ZDV (*p* = 0.02).

### Drug-resistance profiles

At 12 months post-randomisation, 13 out of 14 children with plasma HIV-1 RNA >1000 copies/ml underwent viral resistance genotyping (one viral sequence could not be amplified). Of these 13 children who showed virological failure, 10 (77%) showed at least one major drug-resistance mutation, mainly against NNRTIs (9 out of 13; 69%) or against NRTIs (6 out of 13; 46%) (Table 6). NNRTI-resistance mutations were mainly K103N and Y181C; NRTI-resistance mutations were primarily against 3TC (M184V). Four children with NNRTI-resistant viruses exhibited cross-resistance to second-generation NNRTI treatment with etravirine and rilpivirine. No protease inhibitor resistance was detected. In these 14 children with virological failure at 12 months post-switch, samples prior to ART initiation were analysed retrospectively: three out of seven (43%) from each arm had pre-ART NNRTI-resistance mutations. When comparing the resistance profile 12 months post-randomisation to that observed prior to ART initiation, a non-significant trend towards a higher rate of emerging NNRTI resistance mutations was observed in the EFV arm compared to the LPV arm: five out of six (83%) vs. two out of seven (29%), respectively (Fisher’s exact test, *p* = 0.10). We also noted a high NNRTI-resistance mutation rate prior to ART initiation in the LPV group, reaching 46% (6 out of 13) in children failing at 12 months, even if they were not exposed to PMTCT interventions, probably acquired postnatally via breastmilk from their mother who was initiated on ART before the child. In contrast, five out of six children (83%) in the EFV arm developed a new incident NNRTI-resistance mutation that emerged after the switch. Of note, one child in the EFV arm, prior to ART initiation, harboured a virus resistant to both NRTIs and NNRTIs (mutations M41L, L74I, V108I, M184V, L210W, T215Y, K101E, Y181C, P225H); he/she was born to a mother who had been on ART (AZT-3TC-NVP) for seven years. The four children (two in each arm) exposed to maternal ART through breast milk had new mutations to NNRTI at virological failure.

**Table 6:** Description of the 14 children with virological failure (HIV RNA >1000 copies/mL) after the switch

Number	Country	Arm	HIV-1 subtype	Exposure to prenatal ART, maternal or child PMTCT or postnatal ART via breast milk	HIV RNA at ART initiation (copies/mL)	Mutations at ART initiation			HIV RNA at 12- month post-randomisation (copies/mL)	Mutations at failure post-switch		
						NRTI	NNRTI	PI		NRTI	NNRTI	PI
1	BF	EFV	CRF02_AG	No exposure	19,900,000	None	None	None	6109	None	K103N	None
2	CI	EFV	CRF02_AG	Exposure to child PMTCT (AZT + sdNVP) and postnatal maternal ART (AZT + 3TC + NVP)	242,757	M184V	K103N	None	2091	M184V	K103N, H221Y	None
3	CI	EFV	CRF02_AG	Exposure to maternal PMTCT (AZT) and to child PMTCT (AZT+ sdNVP)	3,573,953	None	None	None	62,866	None	None	None
4	CI	EFV	CRF02_AG	No exposure	1,321,600	None	None	None	102,417	M184V	K103N	None
5	CI	EFV	CRF02_AG	Exposure to maternal PMTCT (AZT + 3TC + NVP) and to child PMTCT (AZT + sdNVP)	3,459,135	None	None	None	1564	Not amplified	Not amplified	Not amplified
6	CI	EFV	CRF02_AG	Exposure to postnatal maternal ART (AZT + 3TC + NVP)	608,578	None	V90I, V179I	None	21,397	M184V	V90I, V179I, K103N	None
7	CI	EFV	CRF02_AG	Exposure to prenatal maternal ART (AZT + 3TC + NVP) initiated 7 years before delivery	12,466,057	M41L, V108I, M184V, L210W, T215Y	Y181C, P225H	None	61,199	M41L, V108I, M184V, L210W, T215Y, L74I	K101E, Y181C, P225H	None
8	BF	LPV/r	CRF02_AG	Exposure to maternal PMTCT (AZT)	7,780,000	None	None	None	7,920,000	None	None	None
9	BF	LPV/r	URF-GK	Exposure to maternal PMTCT (AZT+3TC+NVP) and to child PMTCT (sdNVP) and to postnatal maternal ART (TDF + 3TC + NVP)	4,720,000	None	K103N	None	261,914	None	K103N, Y181C	None
10	BF	LPV/r	CRF02_AG	Exposure to maternal PMTCT (AZT + 3TC + NVP) and child PMTCT (sdNVP)	56,600,000	None	Y181C	None	25,054	None	Y181C	None
11	CI	LPV/r	CRF02_AG	Exposure to child PMTCT (AZT + sdNVP)	254,456	None	None	None	2603	None	None	None
12	CI	LPV/r	CRF02_AG	No exposure	451,590	None	None	None	67,342	M184V	None	None
13	CI	LPV/r	CRF02_CPX	No exposure	915,685	None	K103N, V90I, K101EK	None	3,879,063	None	K103N, V90I, K101EK	None
14	CI	LPV/r	CRF02_AG	Exposure to maternal PMTCT (unknown) and to postnatal maternal ART (AZT + 3TC + LPV/r)	2,710,000	None	None	None	1848	M184V	K103N	None

*ART*, Antiretroviral therapy, *PMTCT* Prevention of mother-to-child-transmission, *NRTI* Nucleoside reverse transcriptase inhibitor, *NNRTI* Non-nucleoside reverse transcriptase inhibitor, *PI* Protease inhibitor, *BF* Burkina Faso, *CI* Cote d’Ivoire, *EFV* Efavirenz, *LPV/r* Lopinavir-boosted ritonavir, *AZT* Zidovudine, *sdNVP* single-dose nevirapine, *3TC* Lamivudine, *NVP* Nevirapine, *TDF*

## Discussion

Our trial provides original findings in the West African context among young HIV-infected children both exposed and not exposed to PMTCT intervention, and virologically suppressed after 12–15 months of a LPV-based ART initiated before 2 years of age. Despite a high-quality follow-up, our randomised trial could not demonstrate non-inferiority for a switch to EFV from LPV/r with regard to the primary outcome (a VL <500 copies/mL) when using both intention-to-treat and per-protocol analyses. Based on completed trial data, the actual statistical power was 67% to detect the planned difference in this outcome. When considering the secondary outcome of HIV RNA <1000 copies/mL, which is commonly used to inform ART switching decisions worldwide, we did demonstrate the non-inferiority of EFV compared to LPV/r. We also showed that switching to a simplified EFV-based therapy in virologically suppressed children below 3 years﻿﻿ of age ﻿is safe, with no deaths and very few severe adverse events, all of which were infectious in nature. However, the pre-switch resistance profiles observed among children with virological failure at 12 months post-switch revealed high rates of NNRTI resistance prior to ART initiation, even in those not exposed to single-dose NVP for PMTCT in both groups. We presume this is due to PMTCT exposure and to postnatal antiretroviral drug exposure through breast milk. New NNRTI mutations were also observed in the EFV arm in children for whom virological suppression failed. We therefore recommend that any simplification switch to EFV needs to be closely monitored.

To date, LPV-sparing strategies have been explored in two paediatric trials in South Africa. These results were firstly reported in the NEVEREST-2 trial, with a switch to a NVP-based ART after first-line ART based on LPV/r [22]. Results from NEVEREST-2 showed that virological suppression at <50 copies/mL at 52 weeks was significantly more common in the NVP “Switch” group (56%) compared to the LPV/r “Stay” group (42%), but remained low overall. However, when the outcome measure was a virological response at <1000 copies/mL, the LPV/r group did significantly better, with 98% suppression vs. 78% for the NVP group *(p* < 0.001). The second protease inhibitor-sparing trial was the NEVEREST-3 trial, published in November 2015, which evaluated a switch to EFV-based ART after LPV/r, similar to our trial [23]. At the time of randomisation, children were on average 4 years of age and had been on treatment for 3.5 years. NEVEREST-3 reported a significantly higher rate of viral rebound to >50 copies/mL in the LPV/r group (*n* = 148) than in the EFV group (*n* = 150), therefore in favour of EFV: 28% of children in the LPV/r experienced an episode of viral rebound vs. 18% in the EFV group. For the second primary endpoint, virological failure (≥1000 copies/mL), there was no significant difference between the groups: 2% of children in LPV/r experienced confirmed virological failure vs. 2.7% of children in the EFV group. The authors concluded that children previously exposed to NVP prophylaxis for PMTCT and initially suppressed on a LPV/r-based regimen did not experience higher rates of viral rebound or virological failure, and can safely switch to an EFV-based regimen [22]. Among the seven children with virological failure, genotyping revealed that three children (42%) had a NNRTI-resistance mutation (K103N) at failure. However, there are several differences between the NEVEREST-3 trial and our trial: all children from NEVEREST-3 were systematically exposed to single-dose NVP for PMTCT and mainly issued from a trial study design; rates of malnutrition (interacting with antiretroviral pharmacokinetics) are lower in South Africa, and viral subtypes differ. Children were younger at ART initiation (median of 9.3 months in NEVEREST-3 vs. 13.7 months in MONOD); at the time of switch, children were 2 years older in NEVEREST-3 compared to MONOD (median 4.3 years vs. 26.8 months, respectively), with, consequently, a 36-month longer duration of viral suppression before randomisation for the switch (3.5 years vs. 12 months); the EFV dosage was higher in the MONOD trial compared to the NEVEREST-3 trial (200 mg/day for a weight of 10–13.9 kg). Our eligibility criteria at randomisation considered children aged <3 years. Currently, EFV is not recommended in children <3 years due to dosing difficulties and the concern for neurological adverse events [24]. However, the FDA approved dosing for children aged 3 months to <3 years as follows: 3.5–5 kg, two 50 mg capsules; 5–7.5 kg, three 50 mg capsules; 7.5–15 kg, one 200 mg capsule (http://www.who.int/hiv/pub/guidelines/arv2013/download/en/index.html). The dosing of EFV in our trial was 25 mg/kg, higher than that recommended [7]. While the minimum age of children in the modelling study of EFV PKs was 2.7 years [16], PK studies are ongoing to identify the appropriate dosage in this population and to provide guidance. Importantly, we did not observe any severe neurological adverse events in our children. Although we were not able to provide data using the <50 copies/mL threshold given the available laboratory assays in our trial, we found similar outcomes to the NEVEREST-3 trial when considering the difference between arms outcome of ≥1000 copies/mL.

We observed high rates of NNRTI resistance in those children who failed to develop virological suppression both before and after failure in both arms. A high frequency of transmitted NNRTI-resistance mutations is expected after exposure to NNRTI-based ART for PMTCT [25]. In addition, we show here that children who have not been exposed to PMTCT are at a very high risk of developing a number of resistance mutations through their exposure to suboptimal doses of maternal antiretroviral drugs through maternal breast milk. Multiclass resistance arises frequently in HIV-infected breastfeeding infants whose mothers are initiated on ART postnatally [26, 27]. These emerging mutations are expected to increase in the context of a large scale-up in maternal ART coverage through the rollout of Option B+. These drug-resistance mutations might negatively impact on future antiretroviral strategies in children who become HIV-infected in a context of limited therapeutic options. However, we did not observe higher rates of failure in the EFV switch arm relative to the LPV/r arm, despite these high pre-ART rates. The accumulation of new NNRTI-resistance mutations was common in children switching to EFV. This suggests that more children who experience virological failure could be expected to accumulate NNRTI-resistance mutations [28]. No resistance mutations to protease inhibitors were detected in our study. Therefore, it is critical that the first-line ART for the growing number of HIV-infected children frequently exposed to suboptimal doses of antiretroviral drugs includes medications with high genetic barriers against resistance.

Finally, it is noteworthy that the implementation of our trial project was operationally complex, resulting in delayed ART initiation at a median age of 14 months, and high mortality in HIV-infected children before ART. During the recruitment period, the national AIDS programmes in both study countries had very low early infant diagnosis (EID) coverage, estimated at 29% in Ouagadougou [29] and 16% in Abidjan [30], mainly due to the post-electoral crisis in 2011. Partially because of this late access to care for infants with HIV infection, the proportion of children alive, in care, and with virological suppression after 12–15 months of LPV was lower than expected: 68% overall, instead of the 90% expected. This led to fewer children with a confirmed undetectable VL at a 3-month interval being eligible for randomisation, affecting the actual statistical power of our trial. We extended the inclusion period to increase the number of children randomised, but for funding reasons, it was not possible to extend this recruitment period beyond February 2013. This highlights that access to EID and early ART before the age of 2 years still remains challenging in real life in 2015. The scale-up of recommended early ART (before 12 months of age) remains one of the major public health challenges in resource-limited settings [29, 31]. Indeed, access to ART in infants requires systematic EID by virological testing in the first weeks of life. EID is complex, expensive and poorly accessible in many African settings [31, 32]. Rollout of EID has been limited, particularly in West African settings where HIV prevalence is low [29, 30, 33]. While LPV-based first-line ART remains the most effective regimen for children aged <3 years, a place for NVP-based first-line ART might be also acknowledged when protease inhibitors are not accessible, even in children exposed to single-dose NVP. Indeed, the ARROW randomised trial recently showed no differences in virological suppression and in resistance to NRTIs or NNRTIs at week 144 between children exposed or unexposed to single-dose NVP prophylaxis receiving NNRTI-based ART and aged <3 years [34].

Several pitfalls should be considered in our trial. The use of a high cut-off to define viral suppression could have led to the accumulation of antiretroviral drug-resistance mutations in the long term. However, this should not have affected the comparability between arms. Also, we were unable to explore the minimal duration of first-line therapy needed to allow a successful switch; our results suggest that it should be at least 12 months. In our trial, the switch to an EFV strategy was conducted only in children with documented virological suppression, leading to a limited generalisability of our approach in settings where HIV VL monitoring is less available, but this was considered to be more ethical to avoid a lack of equipoise in children failing the initial LPV-based therapy. Finally, we were not able to accurately assess adherence using caregiver questionnaires about missing doses during the last 4 days before each monthly visit, but we are currently investigating these data using PK measurements.

## Conclusions

Both the NEVEREST-3 [22] and the MONOD trials provide guidance on the feasibility of switching a child initiated on LPV-based ART to EFV. In our trial, we were unable to conclude on the non-inferiority of EFV compared to LPV at the 500 copies/mL threshold, due to the large confidence interval of the difference, but it was conclusive beyond 1000 copies/mL, similar to the NEVEREST-3 trial. However, given the high rate of NNRTI-resistance mutations at ART initiation in children who failed to develop virological suppression, and the emergence of newly acquired mutations at 12-months post-randomisation (compared to those mutations present at ART initiation) in these children, we would like to be cautious in recommending this switching strategy as a routine public health strategy in low-income countries: this switch strategy needs to be considered only in children with good adherence profiles, and when VL monitoring is available to detect early virological failure after the switch. In settings where ART is delayed and treating young infants remains a significant challenge, with potential exposure to suboptimal doses of maternal antiretroviral drugs through maternal breast milk, it will be particularly crucial to preserve those on suppressive ART of any type, but ideally with a high genetic barrier. In situations where VL monitoring is available before and after the switch, a switch to an EFV-based regimen may be a valuable individualised option in virologically suppressed children with good clinical and adherence profiles. Finally, new formulations of LPV/r that are more palatable and less costly, as well as other future drug options, are still urgently needed to increase ART response in children. Minitab sprinkles formulations of LPV/r are now available but remain poorly palatable [35]. Because treating young children remains challenging in Africa, there is an urgent need to develop formulations appropriate for young children and to make protease inhibitors more widely available, in order to improve the initial response to ART in children. Futures studies are also needed to assess its field response in routine programmes. The potent integrase inhibitor dolutegravir, which has a very low risk for drug-resistance mutations and is currently being formulated for paediatric populations, may represent a valuable option as a first-line therapy or to replace EFV in a switch strategy and needs to be assessed in the future [36].
